# Training AI Models for Aesthetic Facial Evaluation: Focused Review and Framework to Mitigate Homogenizing Bias

**DOI:** 10.2196/95452

**Published:** 2026-06-15

**Authors:** Anisha R Kumar, Lav R Varshney

**Affiliations:** 1 Division of Otolaryngology, Department of Surgery Stony Brook University Stony Brook, NY United States; 2 AI Innovation Institute Stony Brook University Stony Brook, NY United States

**Keywords:** aesthetics, artificial intelligence, fairness, framework, governance, machine learning, medical informatics, model development, plastic surgery, surgery

## Abstract

As artificial intelligence (AI) models become increasingly integrated into facial aesthetic surgery for attractiveness prediction and surgical outcome simulation, their potential to perpetuate bias poses clinical concerns. Current models trained on limited datasets inaccurately evaluate underrepresented populations and risk promoting aesthetic homogenization that conflicts with patient goals of ethnic feature preservation. Drawing on current literature, this paper examines bias across AI development stages in aesthetic facial evaluation. Benchmark datasets such as SCUT-FBP (South China University of Technology—Facial Beauty Prediction) and the Chicago Face Database underrepresent older adults, non-White, and ethnically diverse populations. Training methodologies lack fairness-aware techniques, and evaluation focuses on overall rather than demographic-stratified accuracy. While individual mitigation strategies exist—including balanced datasets, adversarial debiasing, and fairness metrics—no comprehensive framework integrates these approaches across the entire development lifecycle. We propose a 6-pillar framework spanning the AI development lifecycle: (1) diverse data collection with synthetic augmentation, (2) fairness-aware training techniques, (3) complementary fairness metrics with intersectional assessment, (4) explainable AI for clinical transparency, (5) stakeholder engagement, and (6) continuous monitoring. Despite the challenges of maintaining algorithmic standardization and cultural specificity, this framework provides implementation guidance for AI developers, clinicians, and institutions, with principles applicable beyond aesthetic surgery to broader facial analysis applications.

## Introduction

Artificial intelligence (AI) is increasingly integrated into preoperative planning and outcome simulation in facial aesthetic surgery [1,2], typically based on modern machine learning (ML) techniques. Current applications include prediction of attractiveness, simulation of surgical outcomes, and patient assessments [3], offering the potential for objective, standardized aesthetic evaluation [4]. Facial plastic surgeons have preliminarily assessed how established AI-based websites compare to human scoring of facial attractiveness [5], yet standardized and validated AI models for facial aesthetic evaluation have not been established. Before widespread clinical integration, surgeons must understand how these models are trained to ensure accurate, culturally diverse evaluations and avoid perpetuating bias.

The technical challenge of AI model training is compounded by the fact that beauty is a cultural construct rather than a universal fact [6]. Recent large-scale cross-cultural research using geometric morphometrics and Bayesian analysis of 1550 faces from 10 global populations reveals this complexity: distinctiveness (deviation from average facial proportions) negatively affects attractiveness perception universally, and femininity positively influences attractiveness assessments of female faces across all studied populations [7]. However, 2 traditionally emphasized features showed no robust effects: facial symmetry had no significant association with attractiveness ratings, and masculinity did not consistently influence attractiveness judgments of male faces [7]. These findings challenge conventional assumptions about which features are genuinely universal versus culturally variable.

These universal principles interact with culture-specific preferences in complex ways. Skin coloration demonstrates culturally modulated aesthetic judgments: lighter skin tones associate with attractiveness among Chinese observers judging own-ethnicity faces, whereas European observers associate warmer yellow skin tones with attractiveness in Chinese faces [8]. Cross-cultural studies comparing Japanese and American raters reveal that while overall attractiveness ratings correlate across cultures, specific features driving these judgments differ: Japanese raters emphasize raised eyebrows in attractive male faces and smaller mouths in attractive female faces more than American raters [9]. Similarly, considerable cross-cultural agreement exists regarding Vietnamese facial attractiveness, yet Czech European raters associate attractiveness with averageness significantly more than Vietnamese raters [10]. These findings illustrate that while certain structural features (averageness and femininity) demonstrate universal appeal, the relative importance and specific manifestations of these features vary across cultural contexts.

Historically, plastic surgery has relied on Western aesthetic standards [11], but there is growing patient diversity and a shift toward preservation of features that convey ethnic identity [12,13]. An inherent tension, therefore, exists in training AI models: they must account for genuinely universal principles (such as averageness and femininity in female faces) while also recognizing culture-specific preferences and avoiding the imposition of Eurocentric standards on features that demonstrate regional variation [4].

Furthermore, AI models trained on biased datasets may perpetuate narrow beauty ideals and inaccurate representations of patient populations who vary across race, ethnicity, nationality, language, socioeconomic background, gender, and age [14,15], dimensions that interact to produce compounded disadvantage at their intersections, creating a risk that algorithmic recommendations lead to the elimination of distinctive ethnic characteristics and aesthetic homogenization. Culturally responsive AI training frameworks are therefore needed [4]; without them, a gap will persist between the technical capabilities of AI and its ethical implementation in comprehensive patient care [16].

In this paper, we aim to: (1) review AI training methodologies for aesthetic evaluation, (2) examine sources of bias in training, (3) evaluate current practices for mitigating bias, and (4) propose a framework for artificial intelligence/machine learning (AI/ML) training with recommendations for clinical implementation.

## Review of Current Training Methods

Deep learning is a method within AI/ML that uses multilayer neural networks to process large amounts of data and extract complex patterns and features. Within this approach, convolutional neural networks (CNNs) are common for image recognition and processing [16,17]. Such ML models are trained using supervised learning based on human-rated attractiveness scores [18,19], geometric features including symmetry and the Golden Ratio [20], the “rule of thirds” for frontal view analysis [17,20], and extracted facial proportions such as nasofrontal angle, nasolabial angle, and glabella-to-chin angle [17].

Model performance is evaluated using regression tasks, including the Pearson correlation coefficient, which measures the linear relationship between AI-predicted attractiveness scores and human ratings [21,22], and mean absolute error, which measures the average magnitude of prediction errors. These metrics validate how well the AI model replicates human aesthetic judgment. However, recent work in health-related AI has emphasized that evaluation should not rely on task performance metrics alone but also incorporate risk-oriented and context-sensitive assessment frameworks [23].

Beyond attractiveness prediction alone, AI models can simultaneously learn to perform related tasks, including age evaluation, gender identification, ethnicity and race recognition, and facial expression detection [24]. This multitask learning (MTL) approach improves the model’s evaluative capacity across all tasks and develops a more comprehensive understanding of facial features that determine attractiveness. MTL also addresses dataset size limitations (SCUT-FBP [South China University of Technology—Facial Beauty Prediction]: 500 faces; SCUT-FBP5500: 5500 faces) by incorporating auxiliary tasks that provide additional training signals, leveraging natural connections between attractiveness and related attributes [24].

However, recent architectural innovations have moved beyond traditional CNNs to address dataset limitations. Hybrid approaches combining vision transformers and state-space models (such as Mamba) leverage complementary feature extraction capabilities [25]. Vision transformers excel at capturing global facial structure and symmetry through attention mechanisms that process the entire face holistically, while state-space models efficiently model fine-grained local features such as skin quality and texture with linear computational complexity [25].

These architectures use self-supervised pretraining on large, diverse image datasets, followed by task-specific fine-tuning on smaller facial beauty datasets [25]. This transfer learning approach addresses the fundamental challenge that aesthetic datasets, such as SCUT-FBP5500 with 5500 faces, contain orders of magnitude fewer images than typically required for deep learning, while achieving state-of-the-art performance metrics [25].

Several benchmark datasets are currently used to train AI models for attractiveness evaluation. The SCUT-FBP database contains 500 Asian female faces and achieved a Pearson correlation coefficient of 0.8187 between CNN predictions and human ratings [21]. The expanded SCUT-FBP5500 dataset includes 5500 faces with more diverse demographics and achieved correlations of 0.87-0.90 with human evaluators [26]. The Chicago Face Database contains 597 photographs of White and Black male and female individuals aged 17-65 years [27]. Characteristics of each major dataset are detailed in Table 1 [21,26-32].

**Table 1:** Comparison of major facial aesthetic datasets. Characteristics of 8 datasets commonly used to train and evaluate artificial intelligence (AI) models for facial attractiveness prediction, organized by publication year. Datasets were identified from the systematic literature review in this paper. Sample size reflects the most current reported demographic information for each dataset.

Database	Sample size (N); age range (years)	Demographics (race, ethnicity, and gender)	Standardized images	Primary use	Key limitations	Validation metrics
SCUT-FBP (2015) [21]	N=500; not reported	100% Asian 100% Female	Yes (controlled laboratory conditions)	Facial attractiveness prediction benchmark	Small sample Single demographic (Asian females) Limited generalizability	Pearson *r*=0.82
SCUT-FBP5500 (2018) [26]	N=5500; 15-60	73% Asian 27% Caucasian 50% Male 50% Female	No (aggregated from multiple sources)	Multiparadigm facial attractiveness prediction	Limited ethnic diversity (Asian/Caucasian only) Nonstandardized images Variable quality	Pearson *r*=0.87-0.90
Chicago Face Database (2015) [27]	N=597; 17-65	White Black Asian Latino Male Female Percentages unknown	Yes (laboratory photography, standardized conditions)	Research and AI model training across demographics	Small sample May not capture full diversity	Attractiveness ratings by independent raters with interrater reliability; includes mean scores and SDs for multiple attributes
BLINQ (dating site dataset) (not reported) [30]	N>13,000; not reported	Not specified (unknown demographics)	No (collected from dating websites)	CNN^a^ for attractiveness training	Nonstandardized Unknown demographics Selection bias Rating bias	Not reported
Labeled Faces in the Wild (2007) [28]	N=5749; not reported	77.5% Male 83.5% White Remaining unknown	No (captured from online articles/press)	Facial recognition^b^	Severe demographic bias Not designed for aesthetics Nonstandardized	Not applicable (facial recognition dataset)
MEBeauty (2022) [29]	N=2550; not reported	38% White 12% Black 14% Asian 12% Indian 12% Middle Eastern 12% Hispanic 51% Female 49% Male	No (in-the-wild collection)	Facial beauty prediction with ethnic diversity	Small sample Nonstandardized Limited validation Minimal literature usage	Pearson *r* with human ratings; transfer learning validation
FairFace (2021) [31]	N=108,501; 0-70+	50% Female 50% Male 14.3% across major racial groups	No (in-the-wild collection)	Facial recognition^b^	Not designed for aesthetics Nonstandardized No attractiveness ratings	Cross-dataset accuracy; fairness evaluation across demographics
Diversity in Faces (2019) [32]	N=1,000,000; not reported	Not categorized by race/ethnicity—uses objective facial coding schemes	No (in-the-wild collection)	Facial recognition^b^	No race/ethnicity labels No attractiveness ratings	Not reported

^a^CNN: convolutional neural network.

^b^Labeled Faces in the Wild, FairFace, and Diversity in Faces are facial recognition datasets not designed for attractiveness evaluation. They are included because they are widely used as pretraining resources, bias benchmarks, and demographic diversity references in the facial attractiveness AI literature.

As datasets increase in size, standardization becomes increasingly challenging, with many datasets containing mixed lighting conditions, facial expressions, and makeup [30]. A study comparing CNN models trained on the BLINQ dating site database (containing over 13,000 nonstandardized images) vs models trained on BLINQ and then fine-tuned on the standardized Chicago Face Database [27] demonstrated that the 2-step training approach resulted in less variability in attractiveness scores, as facial expressions were shown to confound assessments [30]. These findings underscore the need for purpose-built training systems that can also account for facial expressions.

A critical challenge in current AI/ML training is the feedback loop problem known as “performativity,” where a model’s predictions influence future data distribution [33]. Models trained on specific beauty standards inevitably amplify those standards over time [15]. Bias can be introduced at multiple stages of the AI/ML development lifecycle [34], including targeting bias (defining beauty standards), data acquisition bias (using homogeneous datasets), modeling bias, validation and evaluation bias, and deployment and monitoring bias. Without intentional design for objective assessment, these biases lead to inaccurate and culturally inappropriate aesthetic evaluations.

## Review of Bias Manifestations

Demographic bias is pervasive in facial recognition and aesthetic AI models, resulting in unreliable predictions for underrepresented groups. In a comprehensive evaluation of facial recognition vendor tests, the National Institute of Standards and Technology (NIST) found significant performance variability across different demographic groups and advised users to be aware of these disparities when selecting algorithms [35]. Facial analysis benchmarks demonstrate particularly unreliable predictions for underrepresented demographics, such as females with higher Fitzpatrick skin types [14]. Beyond racial and gender bias, certain age groups, particularly individuals aged older than 60 years, are underrepresented in training data, further compromising accuracy in aesthetic evaluation for these populations [36].

As noted, Eurocentric beauty standards serve as the default framework in AI models [4]. Features flagged as “flaws” may represent valued ethnic traits: broader nasal bridges characteristic of Arab populations are marked for “correction,” while fuller lips common in North African ethnicities are classified as “disproportionate” [4]. Numerous studies have demonstrated that the golden ratio and other neoclassical canons inadequately capture attractiveness across diverse populations [11]. Consequently, AI models built according to singular beauty standards risk inadvertently recommending the westernization of ethnic features without consideration of cultural appropriateness [4].

Regional and cultural aesthetic preferences vary significantly. Patients of Middle Eastern and North African descent often seek nasal tip refinement while preserving other ethnic characteristics such as dorsal height [4,37]. East Asian patients typically prioritize augmentation procedures over reduction [13]. Despite significant cross-cultural variation in motivations, patient expectations frequently include conscious avoidance of “westernized” appearance [37], and high satisfaction correlates strongly with preservation of ethnic features [38].

## Review of Existing Bias Mitigation Approaches

Researchers have developed bias mitigation strategies targeting different stages of AI model development. At the dataset level, initiatives like FairFace have created balanced demographic representations across race, gender, and age categories to address training data imbalances [31]. Synthetic data generation using generative adversarial networks has been proposed to augment underrepresented demographic categories.

Multiple algorithmic interventions have been developed to detect and reduce bias during model training. Adversarial debiasing methods have demonstrated improved fairness outcomes by mitigating bias acquired during data collection [39]. Posttraining corrections, such as centroid fairness loss, enable bias measurement and performance alignment across demographic groups without requiring complete model retraining [40]. Skewness-aware reinforcement learning approaches address data distribution imbalances [41], while techniques like debiasing variational autoencoders adjust sampling probabilities for underrepresented categories [42]. Meta-learning approaches enable models to adapt to regional aesthetic standards with limited culture-specific training data [43].

Bias detection has also advanced through evaluation methodologies. Researchers have documented systematic performance disparities across demographic groups in facial analysis systems. Studies examining intersectional accuracy gaps have revealed that these disparities stem from complex structural factors: for example, poor performance on dark-skinned females in gender classification results not from skin tone itself, but from differences in lip, eye, and cheek structure across ethnicities [44].

## Gaps in Current Approaches

Despite these advances in bias detection and mitigation, critical gaps remain in their application to aesthetic facial evaluation. First, current approaches address bias mitigation at isolated stages rather than across the complete AI development lifecycle. Techniques such as adversarial debiasing [39], centroid fairness loss [40], skewness-aware reinforcement learning [41], and debiasing variational autoencoders [42] have been validated in isolation primarily for facial recognition tasks—where the objective is identity verification—rather than aesthetic evaluation, where subjective cultural beauty standards introduce fundamentally different fairness challenges.

Recent empirical evidence reveals fundamental inadequacies in current mitigation strategies. Dataset diversification efforts like FairFace [31] balance demographic representation yet fail to address annotation bias, the systematic application of culturally-specific aesthetic judgments by raters during labeling [29]. A facial beauty prediction model trained on the multiethnic MEBeauty dataset exhibited significant prediction disparities across ethnic groups (*P*<.001) even when evaluated on balanced data, with only 4.8%-9.5% of intergroup comparisons satisfying distributional parity criteria [45]. More concerning, models demonstrated exacerbated bias on balanced demographic datasets compared to training performance, indicating that current approaches may amplify rather than mitigate societal biases when deployed to real-world populations [45].

Second, no comprehensive framework integrates technical solutions with essential nontechnical components across the AI development lifecycle. Existing approaches lack systematic stakeholder engagement, explainability requirements, and governance structures. While participatory design approaches exist for health care AI broadly [46], aesthetic surgery applications do not systematically involve patients, clinicians, and cultural consultants in model development. Postdeployment monitoring, essential for detecting fairness drift and performance degradation across demographic subgroups [4,35], remains absent from aesthetic AI implementations, with validation protocols testing for bias before deployment rarely incorporated into development pipelines [45].

Third, the field lacks consensus on which fairness metrics are most appropriate for aesthetic contexts. While demographic parity, equalized odds, and equal opportunity are well-defined [47], their application to aesthetic evaluation poses unique challenges: achieving demographic parity may conflict with honoring culturally-specific beauty standards [4], and determining appropriate trade-offs requires stakeholder input that current approaches do not systematically incorporate. These gaps necessitate an integrated framework specifically designed for the unique challenges of bias mitigation in aesthetic facial evaluation.

## Proposed Framework for AI/ML Training in Aesthetic Facial Evaluation

### Overview

To address these gaps, we propose the following framework for the training of AI/ML models in aesthetic evaluation that consists of 6 pillars: data collection and curation, model training methodologies, fairness metrics and evaluation, explainability and transparency, stakeholder engagement, and governance and monitoring.

This framework addresses AI/ML systems used across several distinct aesthetic evaluation tasks, which differ substantially in their fairness stakes and acceptable error thresholds. Attractiveness scoring assigns a rating or ranking to a face and is used primarily for research benchmarking. Preoperative planning uses AI to assess anatomic features and inform surgical approach, where systematic undervaluation of ethnic features could directly influence clinical decision-making. Outcome simulation generates predicted postoperative appearance, where bias has the additional potential to alter rendered ethnicity rather than merely undervalue it, a qualitatively distinct harm addressed further in the framework. Patient counseling involves AI-assisted communication of options, where biased framing may subtly steer patient choices. Fairness requirements and error thresholds should be calibrated to the stakes of the specific use case; the more downstream the application and the more directly it affects patient choice and surgical planning, the more stringent the requirements.

Throughout this framework, recommendations are categorized by their evidence base (Table 2). Practices marked as “established” draw on studies conducted in aesthetic facial evaluation contexts. Practices marked as “adapted” are supported by evidence from adjacent domains, primarily facial recognition, general medical AI, or computer vision, and have been translated to aesthetic evaluation by analogy; these require validation in aesthetic-specific contexts before adoption as standard practice. Practices marked as “proposed” represent conceptual recommendations without empirical validation in any closely related domain and should be treated as research directions.

**Table 2:** Evidence classification for framework recommendations. Summary of bias mitigation practices included in the proposed framework, organized by strength of evidence. Established evidence base indicates practices supported by studies conducted in aesthetic facial evaluation contexts. Adapted evidence base indicates practices supported by evidence from adjacent domains (facial recognition, general medical AI, or computer vision) that have been translated to aesthetic evaluation by analogy and require validation in aesthetic-specific contexts before adoption as standard practice. Proposed evidence base indicates conceptual recommendations without empirical validation in any closely related domain, to be treated as research directions.

Bias source	Pipeline stage	Mitigation strategy	Evidence base	Residual gap
Targeting bias: narrow beauty standard definition	Data collection	Pillar 1: ≥7 ethnic categories Mixed-ancestry probabilistic labeling Continuous morphometric representation	Established	Discrete ethnic categories essentialize group-level patterns Intragroup variation (eg, nationality, socioeconomic background) is rarely captured
Annotation bias: culturally skewed rater judgments	Data collection	Pillar 1: diverse rater recruitment Structured training and calibration Tiered disagreement adjudication Ongoing score audits	Adapted	No validated rater calibration protocol exists for aesthetic evaluation Cultural feature weighting may persist despite diverse panels
Generative adversarial network amplification bias: synthetic augmentation	Data collection	Pillar 1: quality control gate for synthetic images—fairness audit, feature distribution check, and human review	Adapted	Mode collapse and feature exaggeration are documented in generative systems Quality control criteria not validated for aesthetic contexts
Modeling bias: fairness-unaware training	Model training	Pillar 2: adversarial debiasing Centroid fairness loss Skewness-aware reinforcement learning Debiasing variational autoencoder Multitask learning	Adapted	All techniques validated in facial recognition or general computer vision, not aesthetic evaluation Several rest on preprint evidence Combined validation absent
Domain shift bias: train/deploy distribution mismatch	Model training → deployment	Pillar 2: hybrid pretraining on standardized images Fine-tuning on clinical images Domain generalization evaluation prerelease	Proposed	No validated hybrid protocol for aesthetic AI Clinical image variation not systematically characterized
Evaluation bias: aggregate metrics obscure subgroup disparities	Evaluation	Pillar 3: layered fairness metrics with prioritization hierarchy Intersectional assessment Bayesian hierarchical modeling for rare subgroups	Adapted	Thresholds are proposed benchmarks without empirical derivation Metrics can conflict Intersectional sample sizes often insufficient
Explainability gap: black-box outputs in a cultural context	Evaluation → deployment	Pillar 4: Grad-CAM^a^, LIME^b^, SHAP^c^ with required human expert review Geometric/physics-based models as a longer-term goal	Adapted	Explainable AI tools cannot explain why features are culturally valued No method validated for cultural appropriateness verification in aesthetic AI
Human–AI decision bias: clinician interpretation and override	Deployment	Pillar 5 + 6: documentation and audit of AI recommendation override rates by patient demographic Clinician training on implicit bias	Proposed	No empirical data on differential override in aesthetic AI Audit infrastructure absent Accountability for remediation undefined
Deployment bias: commercial systems without governance	Deployment	Pillar 6: disclosure-based accountability for commercial developers FDA SaMD^d^ framework alignment	Proposed	No enforcement mechanism for commercial tools Patient and clinician verification of compliance is currently impossible
Drift bias: postdeployment fairness degradation	Monitoring	Pillar 6: tiered monitoring—continuous process control, quarterly review, annual audit, drift-triggered escalation Designated AI clinical lead	Adapted	Drift thresholds not empirically derived for aesthetic AI Continuous monitoring may not be feasible for community practices

^a^Grad-CAM: gradient-weighted class activation mapping.

^b^LIME: local interpretable model-agnostic explanations.

^c^SHAP: Shapley additive explanations.

^d^FDA SaMD: Food and Drug Administration’s Software as a Medical Device.

### Data Collection and Curation

As a pragmatic baseline informed by existing benchmark datasets, training data should include a balanced representation of at least 7 racial and ethnic categories [31] (White, Black, East Asian, Southeast Asian, Middle Eastern, Latino, and Indian), with additional stratification by gender, age, nationality, and socioeconomic background. This should include multiregional data collection with noted region-specific aesthetic preferences [4,29]. This scheme is explicitly a minimum starting point, not a definitive classification; implementations should adopt more granular schemes as data availability permits. Individuals of mixed ancestry, a rapidly growing population, should be accommodated through multilabel or probabilistic ancestry representation rather than forced assignment to a single category. As the field matures, continuous morphometric representations of facial ancestry, such as principal components of facial geometry derived from diverse reference populations, offer a more biologically-grounded alternative to discrete ethnic labels and should be pursued to reduce the risk of essentialization.

While standardized photographs in a constrained environment are necessary to reduce training inaccuracies from facial expression confounds [30], they introduce a domain-shift risk at deployment: real-world clinical images routinely involve variation in lighting, angle, makeup, and expression that differs systematically from controlled training conditions. To address this, we recommend a hybrid protocol: initial pretraining on standardized images to establish controlled baseline representations, followed by fine-tuning on a curated set of clinically realistic images incorporating documented augmentation strategies—including geometric transformations such as rotation and translation to simulate angle variation, and color space augmentations to simulate lighting variation—to reduce the gap between training and deployment distributions [48]. Explicit evaluation of domain generalization, measuring performance and fairness metric stability across both standardized and nonstandardized image sets, should be required before clinical release.

Even with standardized imaging protocols, acquiring sufficient photographs across all demographic categories remains challenging. Synthetic data generation can bridge these gaps while also protecting privacy, with pretraining gap analysis using established benchmark datasets such as FairFace [31] or Diversity in Faces [32] used to identify demographic imbalances or biases; these have been established in facial attribute classification, though their applicability to aesthetic evaluation requires confirmation. Generative models are themselves trained on real-world data containing existing biases and are susceptible to mode collapse, where the model produces a narrow range of outputs disproportionately representing dominant features, and to hallucination of exaggerated demographic characteristics when conditioned on ethnic labels. These failure modes risk reintroducing the essentialized representations that the framework is designed to prevent. Accordingly, synthetic images must not enter the training set without explicit quality-control steps: adversarial fairness auditing of synthetic outputs to detect feature exaggeration; statistical comparison of synthetic image feature distributions against reference population benchmarks; and human review by the diverse rater panels proposed elsewhere in this framework. Only images passing all 3 criteria should be incorporated.

Furthermore, photograph raters should be recruited from diverse cultural backgrounds to avoid annotation bias [29]. Annotation bias occurs when raters systematically apply their own cultural aesthetic standards to evaluate faces from different backgrounds, but the patterns of bias are complex and do not reduce to simple in-group favoritism. Research demonstrates that rater ethnicity influences which facial features are emphasized—for instance, Chinese observers associate lighter skin tones with attractiveness in own-ethnicity faces, whereas European observers prefer warmer tones in Chinese faces [8], and Japanese raters emphasize raised eyebrows in attractive male faces and smaller mouths in attractive female faces more than American raters [9]. However, cross-cultural studies comparing attractiveness ratings across European, East Asian, and African faces found no strong own-race preference in overall attractiveness judgments [49], indicating that annotation bias operates through subtle feature weighting rather than categorical group favoritism. For example, raters trained predominantly in Western aesthetic ideals might systematically underweight features like broader nasal bridges or fuller lips that are attractive within specific cultural contexts, not because of explicit racial preference but because their cultural training emphasizes different facial proportions. Recruiting ethnically diverse rater panels is therefore essential to ensure balanced representation of aesthetic preferences rather than assuming any single demographic composition will eliminate bias.

### Model Training Methodologies

MTL is central to bias mitigation in AI/ML model training. Models should be simultaneously trained on age, gender, ethnicity, facial expression, and attractiveness ratings to develop a comprehensive and nuanced assessment of faces [36] where feasible. Rater cultural background, region of upbringing, and socioeconomic status should be recorded as covariates to enable analysis of how these dimensions influence annotation.

Several fairness-aware techniques should be implemented during model training, adapted from the facial recognition literature but not yet validated in aesthetic evaluation. Adversarial learning methods [39] should be applied during the training phase to mitigate bias acquired during data collection. Posttraining, centroid fairness loss [40] enables bias measurement and performance alignment across demographic groups without requiring complex model retraining, a significant practical advantage. Skewness-aware reinforcement learning [41] should be used to recognize and adjust imbalances in data distribution or model performance across demographics. Finally, debiasing variational autoencoder [42] can adjust sampling probabilities for underrepresented categories, balancing the effective training data to enable more equitable performance across patient populations.

Beyond these fairness-aware training techniques, AI/ML models can be designed to adapt to regional aesthetic standards for facial evaluation. A meta-learning approach, supported by preliminary evidence from the beauty prediction literature, enables models to “learn how to learn”: models trained on learning tasks from a range of cultures develop the ability to adapt to other cultural preferences more readily [43]. This methodology can account for the subjective nature of beauty perception across cultures and allow customization based on patient population.

While meta-learning enables adaptation to regional standards, the hierarchical structure of aesthetic preferences, with both universal and ethnicity-specific components, suggests opportunities for more sophisticated architectural approaches. Hierarchical Bayesian models could naturally encode this structure through multilevel parameter sharing, where population-level priors capture universal features while group-specific parameters account for cultural variation. Alternatively, the partial invariance framework extends invariant risk minimization by learning features that are invariant within partitions of training environments rather than globally invariant across all environments [50]. Such approaches may encode averageness-related objectives within cultural partitions rather than across the full training distribution, preserving within-group distinctiveness while limiting cross-group homogenization. Universal structural features would be encoded at the population level only when evidence supports genuine cross-cultural validity, while regionally variable features remain governed by culture-specific parameters. Efficient multigroup equivariant techniques that address intersectional fairness across combinations of protected attributes, such as ethnicity and gender, may offer additional methodological directions [51]. However, these techniques have not yet been validated for subjective aesthetic judgments, and their applicability to facial evaluation remains an open empirical question.

Until such validation is established, these advanced architectural approaches should be considered active research directions rather than recommended clinical components. Several debiasing techniques in this section also rest on preprint or single-study evidence whose reproducibility has not been independently established; responsible clinical adoption requires, at a minimum, prospective studies demonstrating improvement in prespecified fairness metrics across multiple independent ethnic groups, independent reproducibility on held-out datasets, and direct comparison against uncontrolled baseline systems under clinically realistic conditions.

### Fairness Metrics and Evaluation

A comprehensive fairness evaluation system integrates group fairness metrics, performance standards, and intersectional assessment. This layered approach prevents the common problem of achieving fairness on average while still having significant disparities in specific subpopulations, which is particularly critical in aesthetic facial evaluation, where cultural considerations vary significantly across intersectional identities.

Fairness metrics are chosen based on the clinical application to facial evaluation. Group fairness metrics measure and reduce bias to ensure that models evaluate different demographic groups equitably [47]. Demographic parity catches outcome bias by ensuring that the models’ aesthetic ratings are independent of ethnicity and equal across different demographic groups. Since the clinical utility of these models requires accuracy as well, equalized odds catches accuracy bias by ensuring that the models perform equally well across all demographic groups, with false positives and false negatives occurring equally. Additionally, the evaluation system should include equal opportunity metrics as an option for clinicians when identifying positive outcomes is the priority and false positives are not an issue. In an aesthetic evaluation scenario, equal opportunity focuses on not missing attractive features while being more flexible about possible overestimation. However, different fairness metrics can oppose each other, which makes it axiomatically impossible to align all of them simultaneously.

We propose the following benchmarks as starting points for community debate and empirical refinement, not as validated thresholds: cultural concordance scores of at least 80%, as reviewed by regional expert review panels [4], feature recognition accuracy of at least 95% [4], and demographic parity in prediction accuracy with no more than 5% variance across groups. These figures represent reasonable aspirational targets informed by analogous fairness benchmarks in other clinical AI applications but require prospective validation in aesthetic evaluation contexts before adoption as standards.

Because fairness metrics will conflict in practice, a decision hierarchy is necessary. Consider the following scenario: a model achieves demographic parity (equal average attractiveness ratings across ethnic groups) but does so by consistently overpredicting attractiveness for underrepresented groups while underperforming on fine-grained feature recognition for those same groups. Demographic parity is satisfied; equalized odds are not. In this scenario, we recommend prioritizing equalized odds because accuracy parity across groups is a prerequisite to the clinical utility of the tool. A model that fails to accurately recognize features in specific ethnic groups cannot serve those patients equitably, regardless of average score distributions. Equal opportunity metrics should then be applied as a secondary check, specifically where the clinical priority is avoiding false negatives, for example, ensuring that attractive features in underrepresented populations are not systematically missed.

This hierarchy assumes deployment in a pluralistic patient population. In settings where the clinical population is demographically homogeneous, the relevant fairness question shifts: the priority becomes within-population accuracy and avoidance of intragroup bias, rather than cross-group parity. In such contexts, fine-tuning on locally representative data may be both technically appropriate and ethically indicated, provided that the resulting model is transparently scoped to its intended deployment population and not generalized beyond it. A model developed and validated for a specific national or regional context, for example, a system trained primarily on Korean patients for deployment in South Korea, should be evaluated against locally derived aesthetic norms and demographic distributions, and its scope of applicability documented accordingly.

Since the above-mentioned techniques evaluate for biases for individual criteria such as race or age, an intersectional assessment should be introduced into model training to evaluate overlapping biases that may compound. In aesthetic facial evaluation, intersectional bias due to the aggregation of multiple social identities, such as race, ethnicity, nationality, socioeconomic background, gender, and age, can incorrectly influence outcomes and may perpetuate stereotypes [14]. An intersectional assessment provides the framework to apply metrics and standards across complex, overlapping demographic categories such as Black women or older Asian men.

Since individuals hold multiple overlapping identities simultaneously, each with associated social norms and expectations, aesthetic preferences cannot be understood by analyzing demographic categories in isolation, a theoretical foundation reinforcing that intersectional assessment addresses the fundamental mechanism through which identity influences perception [52].

Practically, however, current dataset sizes preclude stable estimates across all intersectional combinations, necessitating a prioritization hierarchy. High-risk intersections with documented performance disparities—such as older women with higher Fitzpatrick skin types—should be evaluated as the primary tier. Compositional approaches offer a promising avenue for addressing certain intersectionality challenges more tractably: multigroup equivariant network designs that use product groups can provide fairness guarantees across intersectional demographic combinations with computational complexity proportional to the sum rather than the product of group sizes, as demonstrated in natural language generation debiasing tasks [51]. Second, Bayesian hierarchical modeling of rare subgroups enables partial pooling of statistical strength from related intersections, providing more stable estimates for low-frequency cells; this approach ties naturally to the hierarchical architectures recommended in the Model Training Methodologies section. Third, multitask regularization can share statistical strength across related intersectional categories during training. Intersectional combinations not covered by the primary tier should be explicitly designated for future work rather than omitted without acknowledgment.

### Explainability and Transparency

Explainability and transparency are prerequisites for trustworthy AI/ML systems in clinical use. Three different techniques adapted from general medical AI would be beneficial for training models used in aesthetic facial evaluation: gradient-weighted class activation mapping (Grad-CAM), local interpretable model-agnostic explanations (LIME), and Shapley additive explanations (SHAP) [53]. Grad-CAM provides visual and spatial explanations through heatmaps that highlight which facial regions contribute most to a model’s aesthetic predictions. Clinicians can identify which anatomic feature the model prioritizes and can also verify that the model emphasizes culturally appropriate features rather than defaulting to Eurocentric beauty standards. LIME provides case-specific explanations of a model’s individual predictions by showing which features influenced that specific assessment. This technique is model-agnostic and therefore flexible and versatile, meaning it can be applied to any AI/ML architecture—whether convolutional neural networks, transformer models, or future technologies—making it adaptable as the field evolves. SHAP provides information about how each evaluated feature contributes to the specific output of a model. It can explain individual predictions and provide a global overview of which features are most important in a dataset. The practical value of SHAP-based interpretation has also been demonstrated in surgical predictive AI, where feature-level explanation was used to identify key perioperative risk factors and improve transparency of model behavior for clinical decision-making [54]. While these methods continue to evolve with ongoing algorithmic refinements, these tools are useful but not sufficient for verifying cultural appropriateness in aesthetic evaluation. They identify which facial regions or features influence a model’s output, but do not explain why those features are aesthetically valued within a specific cultural context, which is the central interpretive question this framework is designed to address. Human expert review by culturally knowledgeable clinicians is, therefore, a required complement to explainable AI (XAI) output, not an optional one.

Intrinsically interpretable approaches, which incorporate domain knowledge directly into model architecture rather than applying post hoc explanation methods, represent a more direct path toward clinical-grade cultural interpretability. For facial aesthetic evaluation, this includes explicitly encoding geometric relationships (such as facial proportions, angles, and distances) as structured features within the model, and physics-based models that incorporate established anatomical principles and morphometric relationships.

Transparency standards should ensure that stakeholders have access to essential information about model development and validation. Transparency requirements for AI/ML training models should include the documentation of training data demographics and model architecture, interpretable explanations for aesthetic assessments, and disclosure of identified biases and performance disparities across demographics.

For generative outcome simulation systems specifically, XAI tools must address an additional interpretive requirement: clinicians should be able to verify that simulated postoperative appearances reflect only the intended surgical modifications and do not introduce ethnically incongruent features as artifacts of model bias. This requires comparison of presimulation and postsimulation facial geometry at the feature level, which current Grad-CAM and LIME implementations are not designed to provide; bespoke evaluation protocols for generative systems are needed.

### Stakeholder Engagement and Participatory Design

The development of AI models should involve AI developers, patients, clinicians, cultural consultants, and ethicists as equal partners from the conceptualization stage through implementation and postdeployment monitoring [46]. This cocreation process ensures that diverse perspectives shape decision-making at every stage, from selecting training datasets to interpreting model outputs to refining algorithms based on real-world performance. Workshops and iterative feedback sessions should be conducted throughout the development lifecycle to gather input on critical questions such as which facial features should be prioritized for analysis, how to define culturally appropriate aesthetic outcomes, and whether model recommendations align with patient values and clinical judgment. Participant recruitment should deliberately include patients who identify across multiple marginalized dimensions simultaneously, such as older immigrant women from non-Western countries, as their aesthetic priorities and experiences of algorithmic bias are likely to differ from those captured by single-axis demographic sampling. It is vital to recognize and address potential power dynamics to ensure that underrepresented stakeholders’ voices are valued equally alongside technical experts and established institutions.

Regional expert review panels convened to evaluate cultural concordance must be constituted with explicit accountability requirements. Panel composition should include representatives from diverse geographic regions within each cultural community, not only urban or elite centers, and should reflect variation in socioeconomic background, age, and gender. Selection criteria should be documented and publicly disclosed. Formal mechanisms for recording minority and dissenting views are required; panel reports should distinguish consensus from majority positions and preserve dissenting opinions for review. Periodic panel rotation and external audit of panel composition guard against the entrenchment of a single institutionalized aesthetic perspective. These panel requirements must be operationalized through rigorous rater training and calibration protocols.

Rater training and calibration protocols are essential to annotation quality. Before scoring, raters should complete structured training that includes: an orientation to the study’s cultural equity goals; exposure to diverse face exemplars across all demographic categories to be rated; and explicit instruction to evaluate attractiveness according to within-group cultural standards rather than a universal ideal. Calibration should be conducted using a standardized set of anchor images—rated in advance by a culturally matched expert panel—against which individual rater scores are benchmarked. Raters whose scores diverge systematically from calibration anchors by more than a prespecified threshold (for example, mean absolute deviation greater than 1.0 on a 10-point scale) should receive additional training before contributing to the primary dataset. For images where rater scores span more than 3 points on a 10-point scale, the image should be flagged for adjudication by a culturally matched expert reviewer rather than resolved by averaging. Averaged scores obscure genuine aesthetic disagreement that may itself be informative about cultural variation. Ongoing audit of rater score distributions by demographic subgroup should be conducted throughout data collection to detect systematic drift in individual rater calibration.

A human-centered design framework positions clinicians and patients as essential collaborators while respecting their cultural contexts and prior experiences. Patients who have undergone aesthetic procedures provide experiential knowledge about how cultural identity influences aesthetic goals, what features they sought to preserve vs modify, and how algorithmic recommendations might have impacted their decision-making. This bidirectional learning process builds cultural competency across all stakeholders and creates the foundation for AI systems that serve diverse populations equitably.

Additionally, informed consent architecture is a prerequisite for ethical dataset development. Individuals depicted in training images must provide explicit consent for secondary use of their facial photographs in AI training, with the right to withdraw consent and have their images removed from future training cycles. This requirement applies regardless of whether images are sourced from clinical records, publicly available datasets, or social media platforms, and must account for jurisdiction-specific biometric privacy regulations and state-level statutes. However, consent withdrawal raises a technically significant challenge: once a model is trained on data, removing a data point’s influence from a deployed model without full retraining is computationally costly. The emerging field of machine unlearning addresses this problem through methods such as approximate unlearning and influence function-based data removal, though these techniques have not yet been validated or operationalized in medical AI governance contexts. Until practical machine unlearning protocols are established for clinical AI, consent frameworks should, at a minimum, guarantee removal from future retraining cycles and document this limitation transparently as a residual risk. Raters whose aesthetic judgments become training labels should similarly provide informed consent, be compensated equitably, and retain the right to withdraw their ratings from the dataset. Evolving Food and Drug Administration (FDA) guidance on training data provenance under the Software as a Medical Device (SaMD) framework should be monitored for additional requirements as it develops. Documentation of consent procedures, including the current technical limitations of consent withdrawal from deployed models, should be included in the transparency disclosures required elsewhere in this framework.

### Governance and Continuous Monitoring

A governance structure for AI models should be based on multidisciplinary committees including clinicians, ethicists, data scientists, and patient representatives. This allows for ongoing ethical review with diverse stakeholder input and mandates human oversight for all AI-driven recommendations, requiring clinician review of model outputs before they inform patient consultations or treatment planning. To ensure regulatory compliance, developers should align with the FDA’s SaMD framework [55] and remain aware of evolving federal regulations for AI in health care.

Bias in the human–AI decision system extends beyond model development to the point of clinical use. Even a well-calibrated, fairness-aware model can produce inequitable outcomes if clinicians or institutions apply its outputs selectively or inconsistently across patient groups. Research on physician implicit bias [56] suggests that providers may differentially override algorithmic recommendations based on patient demographics, accepting recommendations for patients who resemble the provider’s implicit reference population while discounting them for others. Governance structures should include mandatory documentation of AI recommendation acceptance or rejection by patient demographic category, regular review of override rates stratified by patient race, ethnicity, gender, age, and language, and structured clinician training on the mechanisms of implicit bias in AI-assisted decision-making. Where systematic override disparities are detected, the governance committee should determine whether the source is model error for specific subgroups or clinician bias in interpretation. Fairness in aesthetic AI, therefore, requires monitoring both algorithmic outputs and human responses to those outputs.

Let drift be a statistically significant change, exceeding prespecified control limits, in 1 or more of the following: prediction accuracy by demographic subgroup, fairness metric values (demographic parity, equalized odds, cultural concordance), or the distribution of model inputs relative to the training distribution. Demographic-subgroup-specific degradation that does not affect overall accuracy is a particularly important drift signal, as aggregate metrics can mask emerging disparities. Institutional responsibility for drift response should be assigned explicitly at deployment: a designated AI clinical lead bears primary responsibility for reviewing automated alerts, convening the multidisciplinary governance committee, and authorizing remediation. Triggered remediation actions should follow a tiered protocol keyed to severity: minor drift triggers increased monitoring frequency and a targeted data audit; moderate drift triggers model recalibration or posttraining correction without full retraining; severe drift, including any demographic subgroup falling below institutionally defined minimum performance thresholds, triggers suspension of AI-assisted outputs for affected use cases pending full model retraining and revalidation. The specific thresholds delineating these severity tiers should be defined prospectively by each deploying institution based on clinical context, use case stakes, and available monitoring infrastructure, rather than adopted from universal benchmarks for which no empirical basis currently exists in aesthetic AI. All drift events and remediation actions should be documented in an institutional AI governance log and reported in periodic transparency disclosures.

Postdeployment monitoring of the AI/ML models should include continuous tracking of prediction accuracy, fairness metrics across demographic groups, and concordance with clinical judgment. A tiered monitoring approach is recommended. Continuous monitoring using statistical process control on prespecified fairness metrics, with automated alerts when metrics exceed defined control limits described above, provides the first line of detection. Quarterly structured reviews should assess fairness metric trends and flag emerging disparities for clinical review. An annual deep audit evaluates model architecture, training data composition, rater panel diversity, and alignment with updated regulatory guidance. Where continuous monitoring infrastructure is not feasible, drift-triggered audits, initiated automatically when prediction distributions shift beyond a prespecified threshold, represent a minimum acceptable alternative. The appropriate monitoring intensity scales with deployment volume: high-volume systems serving diverse patient populations require continuous monitoring; lower-volume or single-institution implementations may operate on a quarterly plus annual cycle with drift-triggered escalation. When audits identify performance disparities exceeding 5% variance across demographic groups, retraining (or appropriate posttraining) should be initiated. Feedback loops integrating clinician and patient input, as well as regular retraining cycles incorporating new, diverse data, allow for ongoing model improvement.

Ultimately, this framework is directed primarily at academic and institutional developers and is intended as aspirational guidance and input to regulatory deliberation. Most currently deployed aesthetic AI tools are commercial, including consumer-facing filters, practice-management platforms, and direct-to-consumer assessment applications, and fall outside the scope of institutional governance structures. We recommend that regulatory bodies consider disclosure-based accountability as a lighter-touch regulatory instrument: commercial developers would publicly document which framework components their systems satisfy, analogous to transparency requirements in other regulated industries, enabling clinicians and patients to assess compliance. We acknowledge that even this approach requires formal regulatory action and cannot be implemented through voluntary adoption alone. Long-term, enforcement mechanisms aligned with the FDA’s evolving SaMD framework and equivalent international regulations will be necessary to extend these standards to commercial systems.

## Limitations and Unresolved Challenges

This framework addresses critical biases in aesthetic AI training but faces implementation challenges in operationalizing the distinction between universal and culture-specific aesthetic features. While empirical evidence establishes that certain features (averageness, femininity in female faces) demonstrate cross-cultural appeal, whereas others (skin coloration emphasis, specific feature preferences) vary by cultural context, translating this nuance into algorithmic systems remains complex. Hierarchical approaches, including hierarchical Bayesian models, partial invariance, and multigroup equivariant techniques [50,51,57], theoretically offer a middle ground by encoding universal structural principles in base layers while allowing culture-specific parameters for regionally variable features. However, practical deployment carries risks: explicitly categorizing training data by ethnicity may essentialize group-level patterns, calcifying what constitutes “Asian beauty” or “African beauty” rather than honoring individual variation within cultural communities. Moreover, broad ethnic categories obscure meaningful contextual differences; for instance, North Korean, South Korean, Korean-American, and Korean-Canadian individuals may hold divergent aesthetic preferences despite shared ethnic heritage, yet training data rarely capture this granularity. The framework’s emphasis on continuous monitoring and stakeholder feedback provides mechanisms to detect such unintended consequences, but cannot eliminate these tensions entirely.

Technical limitations constrain practical implementation. As noted, fairness metrics can mathematically conflict, and when improving performance for one demographic group worsens outcomes for another, the framework provides insufficient guidance on prioritization. Intersectional assessment becomes computationally prohibitive when evaluating all meaningful combinations of race, age, gender, and other attributes, requiring sample sizes that may not exist for rare intersectional categories. Most proposed techniques have been validated in isolation rather than as an integrated system, creating uncertainty about their combined effectiveness.

The framework may create unintended harms despite ethical intentions. Formalizing cultural aesthetic standards into training data risks reifying what should remain individually variable. Overreliance on AI-assisted evaluation may affect clinicians’ ability to make nuanced judgments. The resource-intensive requirements, including multidisciplinary committees, annual audits, and continuous monitoring, may be feasible only for well-funded institutions, potentially widening disparities between elite centers and community practices that either avoid AI tools entirely or use inadequately validated commercial systems.

Despite these significant limitations, this framework represents the most viable approach to addressing documented harms in current systems. Culture-neutral algorithms trained on predominantly Western datasets demonstrably perpetuate Eurocentric beauty standards and generate systematically higher error rates for underrepresented populations [4,14,35]—creating risk that algorithmic recommendations may conflict with patient goals of ethnic feature preservation [37,38]. Intentionally encoding cultural awareness during training is preferable to allowing implicit Western bias to persist unchecked, and the framework’s emphasis on continuous monitoring and stakeholder engagement provides mechanisms for identifying and correcting unintended consequences as they emerge, making this an iterative rather than static solution. Bias mitigation in aesthetic AI remains an evolving challenge requiring ongoing research, stakeholder dialogue, and willingness to revise approaches as evidence accumulates.

## Implementation Considerations

Implementing this framework requires strategic approaches to address inherent complexities in developing fair and accurate aesthetic evaluation systems. A phased implementation strategy allows institutions to prioritize components based on available resources, with initial phases focusing on data diversification and basic fairness metrics before advancing to sophisticated techniques such as meta-learning and intersectional assessment.

Addressing data limitations requires combining synthetic data generation, transfer learning methods, and multi-institutional collaboration. Pooling datasets across institutions expands demographic coverage while distributing resource burdens and facilitating industry standards for dataset requirements, fairness thresholds, and validation protocols. Federated learning, established in medical imaging and adapted for aesthetic AI applications, provides a privacy-preserving framework that enables multi-institutional and multinational collaboration while maintaining HIPAA (Health Insurance Portability and Accountability Act) compliance. In this approach, each institution trains models on its local dataset without sharing raw patient images; only model parameters are transmitted between sites, addressing both regulatory requirements and the need for demographically diverse training data. This decentralized architecture has been successfully demonstrated in medical imaging applications, showing that models trained via federated learning can achieve comparable or superior performance to centralized training while preserving patient privacy [58]. However, vanilla federated learning does not guarantee privacy: gradient leakage, membership inference attacks, and model inversion techniques can reconstruct sensitive features of training images from transmitted model parameters. Clinically deployable federated systems, therefore, require additional mitigations: differential privacy limits individual-level information leakage, secure aggregation protocols ensure that parameter updates are aggregated without exposing individual site contributions, and homomorphic encryption may be warranted in high-risk deployments involving sensitive biometric data. HIPAA compliance of a federated system depends on these additional safeguards, not on the federated architecture alone, and implementers should document which mitigations are in place as part of their data governance and regulatory submissions.

Multisite data acquisition requires harmonization protocols that go beyond technical compatibility. Participating institutions should adopt a shared data dictionary defining demographic subgroup categories, attractiveness rating scales, and imaging standards before data collection begins; naive pooling of heterogeneous datasets across sites can amplify distributional asymmetries and yield biased estimators, and post hoc harmonization of inconsistently defined variables compounds this risk [59]. Subgroup definitions should be governed by a standing dataset governance committee with representation from each participating institution and from community members of the populations being represented; this committee should have authority to revise subgroup definitions as evidence about their validity accumulates. Long-term dataset maintenance requires designated institutional roles: a data steward responsible for tracking consent status and honoring withdrawal requests, a technical curator responsible for version control and documentation of any dataset changes, and a scientific lead responsible for periodic assessment of whether the dataset’s demographic composition remains representative of the clinical population it is intended to serve. Datasets should be versioned explicitly so that fairness audits can be traced to the data on which a model was trained. Multisite data sharing agreements should specify data retention limits, destruction protocols, and procedures for incorporating newly consented data into existing pipelines without reintroducing batch effects.

The selection of fairness metrics presents practical trade-offs between comprehensiveness and computational efficiency. Given that simultaneous optimization is mathematically impossible, developers should understand that different metrics detect distinct types of bias. For facial aesthetic evaluation, using multiple complementary metrics is essential: demographic parity identifies whether the model systematically undervalues certain demographic groups, equal opportunity detects failures to recognize attractive features in underrepresented populations, and equalized odds ensures accuracy parity across all groups. While resource constraints may require prioritizing certain metrics during initial implementation, comprehensive evaluation across multiple fairness dimensions should remain the long-term goal. Metric prioritization should be calibrated to the clinical use case. For example, a preoperative planning tool used across a demographically diverse practice should prioritize equalized odds, ensuring that error rates in feature recognition are equivalent across ethnic groups, because differential accuracy directly affects surgical decision-making. By contrast, an outcome simulation tool used in patient counseling might prioritize equal opportunity, ensuring that attractive features in underrepresented groups are not systematically missed or underrendered, because the primary harm is failure to surface positive options rather than differential error rates. A research benchmarking tool, with lower direct clinical stakes, might accept demographic parity as a sufficient initial standard while longitudinal validation data are collected.

## Conclusion

**Figure 1:**
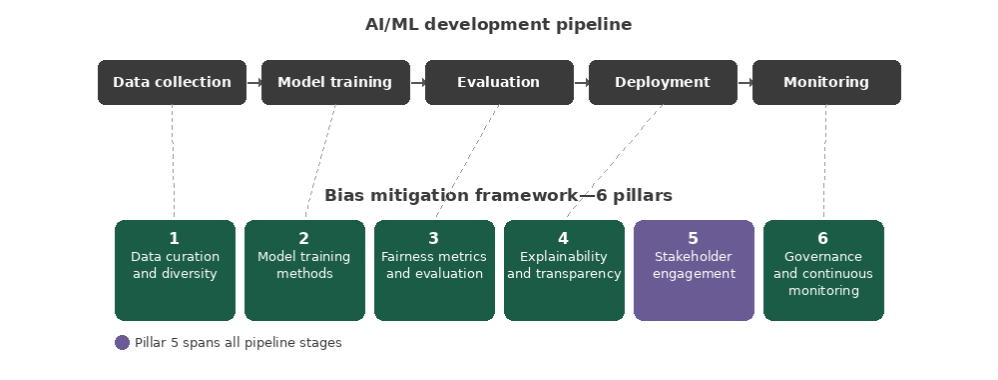
The 6-pillar framework for bias mitigation in artificial intelligence/machine learning (AI/ML) models for aesthetic facial evaluation. Visual overview of the proposed framework spanning the full AI development lifecycle. The six pillars are: (1) diverse data collection with synthetic augmentation, (2) fairness-aware training techniques, (3) complementary fairness metrics with intersectional assessment, (4) explainable AI for clinical transparency, (5) stakeholder engagement and participatory design, and (6) governance and continuous monitoring. Arrows indicate the interdependent nature of the pillars across the development lifecycle.

The integration of AI/ML into aesthetic facial evaluation presents both opportunity and risk. Without intentional intervention, algorithmic systems will perpetuate Eurocentric beauty standards, generate higher error rates for underrepresented populations, and risk aesthetic homogenization. This framework provides comprehensive bias mitigation through 6 interconnected pillars, from diverse data collection and fairness-aware training to XAI, stakeholder engagement, and continuous monitoring (Figure 1).

The framework mitigates the tension between universal aesthetic principles and cultural specificity rather than fully resolving it; the mathematical conflict between optimizing for averageness and preserving ethnic distinctiveness remains an open research problem requiring empirical validation of hierarchical architectural approaches. Within these constraints, the framework positions diverse stakeholders, including patients, clinicians, cultural consultants, and ethicists, as essential collaborators whose input shapes algorithm development and refinement.

Significant challenges remain: tensions between algorithmic objectivity and cultural subjectivity may not be fully resolved, fairness metrics may conflict, and resource-intensive implementation may widen institutional disparities. Despite these limitations, this framework represents a necessary step toward ethical AI development in aesthetic medicine, providing actionable guidance for developers, clinicians, and institutions committed to equitable care.

For this framework to move from proposal to validated practice, concrete validation end points must be defined. Prospective comparative studies should measure prespecified fairness metrics, such as demographic parity, equalized odds, and cultural concordance, in framework-compliant systems versus uncontrolled alternatives. Patient-reported outcomes measuring satisfaction with ethnic feature preservation should serve as a primary clinical end point, given that algorithmic recommendations ultimately affect patient goals and identity. Clinician agreement studies should assess whether XAI-explained outputs align with expert aesthetic judgment across demographic subgroups. Finally, longitudinal drift detection benchmarks should evaluate whether fairness gains are maintained as models are retrained on new data. Randomized deployment designs, in which framework-compliant and noncompliant systems are compared in parallel with patient consent, would provide the strongest evidence but raise practical and ethical challenges requiring dedicated methodological attention.

As AI capabilities advance, ongoing research must address operationalizing cultural appropriateness, validating integrated bias mitigation techniques, and ensuring technological progress serves patient-centered, culturally responsive care. This broader implementation challenge is consistent with recent work emphasizing that AI adoption in health care requires structured implementation pathways and explicit risk mitigation strategies rather than relying on technical advancement alone [60]. These principles extend beyond aesthetic surgery to any facial analysis AI application, establishing foundations for fair and transparent algorithmic systems across diverse clinical contexts.

## Data Availability

Data sharing is not applicable to this article as no data sets were generated or analyzed during this study.

## Abbreviations

AI/ML: artificial intelligence/machine learning
AI: artificial intelligence
CNN: convolutional neural network
FDA: Food and Drug Administration
Grad-CAM: gradient-weighted class activation mapping
HIPAA: Health Insurance Portability and Accountability Act
LIME: local interpretable model-agnostic explanations
ML: machine learning
MTL: multitask learning
NIST: National Institute of Standards and Technology
SaMD: Software as a Medical Device
SCUT-FBP: South China University of Technology—Facial Beauty Prediction
SHAP: Shapley additive explanations
XAI: explainable AI
